# Survey and Evaluation of Hypertension Machine Learning Research

**DOI:** 10.1161/JAHA.122.027896

**Published:** 2023-04-29

**Authors:** Clea du Toit, Tran Quoc Bao Tran, Neha Deo, Sachin Aryal, Stefanie Lip, Robert Sykes, Ishan Manandhar, Aristeidis Sionakidis, Leah Stevenson, Harsha Pattnaik, Safaa Alsanosi, Maria Kassi, Ngoc Le, Maggie Rostron, Sarah Nichol, Alisha Aman, Faisal Nawaz, Dhruven Mehta, Ramakumar Tummala, Linsay McCallum, Sandeep Reddy, Shyam Visweswaran, Rahul Kashyap, Bina Joe, Sandosh Padmanabhan

**Affiliations:** ^1^ School of Cardiovascular and Metabolic Health University of Glasgow Glasgow United Kingdom; ^2^ Mayo Clinic Alix School of Medicine Rochester MN; ^3^ Center for Hypertension and Precision Medicine, Department of Physiology and Pharmacology University of Toledo College of Medicine and Life Sciences Toledo OH; ^4^ Institute of Genetics and Cancer University of Edinburgh Edinburgh United Kingdom; ^5^ Lady Hardinge Medical College New Delhi India; ^6^ Department of Pharmacology and Toxicology, Faculty of Medicine Umm Al Qura University Makkah Saudi Arabia; ^7^ College of Medicine Mohammed Bin Rashid University of Medicine and Health Sciences Dubai UAE; ^8^ Department of Internal Medicine TriStar Centennial Medical Center, HCA Healthcare Nashville TN; ^9^ School of Medicine Deakin University Geelong Australia; ^10^ Department of Biomedical Informatics University of Pittsburgh Pittsburgh PA; ^11^ Department of Anesthesiology and Critical Care Medicine Mayo Clinic Rochester MN

**Keywords:** artificial intelligence, hypertension, machine learning, reporting quality, Hypertension, High Blood Pressure, Machine Learning

## Abstract

**Background:**

Machine learning (ML) is pervasive in all fields of research, from automating tasks to complex decision‐making. However, applications in different specialities are variable and generally limited. Like other conditions, the number of studies employing ML in hypertension research is growing rapidly. In this study, we aimed to survey hypertension research using ML, evaluate the reporting quality, and identify barriers to ML's potential to transform hypertension care.

**Methods and Results:**

The Harmonious Understanding of Machine Learning Analytics Network survey questionnaire was applied to 63 hypertension‐related ML research articles published between January 2019 and September 2021. The most common research topics were blood pressure prediction (38%), hypertension (22%), cardiovascular outcomes (6%), blood pressure variability (5%), treatment response (5%), and real‐time blood pressure estimation (5%). The reporting quality of the articles was variable. Only 46% of articles described the study population or derivation cohort. Most articles (81%) reported at least 1 performance measure, but only 40% presented any measures of calibration. Compliance with ethics, patient privacy, and data security regulations were mentioned in 30 (48%) of the articles. Only 14% used geographically or temporally distinct validation data sets. Algorithmic bias was not addressed in any of the articles, with only 6 of them acknowledging risk of bias.

**Conclusions:**

Recent ML research on hypertension is limited to exploratory research and has significant shortcomings in reporting quality, model validation, and algorithmic bias. Our analysis identifies areas for improvement that will help pave the way for the realization of the potential of ML in hypertension and facilitate its adoption.

**Nonstandard Abbreviations and Acronyms**
ML: machine learning

**Clinical Perspective**

**What Is New?**

- The number of hypertension research studies employing machine learning (ML) is increasing quickly, but, as in other clinical domains, there has been almost no translation into clinical practice, and there are concerns about the robustness and generalizability of ML models applied to diverse populations, as well as the quality and reporting of ML methods and results in the clinical research.
- Frameworks are now being developed for conduct and reporting of clinical ML research.
- Because of the variety of input data and the customizability of ML methods, disease‐ and domain‐specific recommendations are to be required for ML.

**What Are the Clinical Implications?**

- Our analysis of recent hypertension ML publications identifies areas for improvement in reporting, which should inform and support hypertension researchers who are using or planning to use ML.
- This study will help clinicians evaluate commercial ML tools for clinical use effectively and thus minimize patient harm and improve clinical service.

Recent advances in computational power and the availability of larger and more comprehensive medical data sets have led to an increase in machine learning (ML) in clinical research, which could transform health care. Despite the rapid increase in research and evidence that ML models outperform clinicians in areas such as arrhythmia detection and clinical image processing, the actual impact on health care has been limited.^1^, ^2^, ^3^ Hypertension is the single most important modifiable risk factor worldwide, causing nearly 10 million deaths annually in both high‐ and low‐income countries. The management of hypertension, from screening to diagnosis to treatment, presents a number of obstacles that call for transformational solutions in which ML may play a role.^4^, ^5^ In fact, the number of research studies employing ML is increasing quickly, but, as in other clinical domains, there has been almost no translation into clinical practice. A solid understanding of the clinical domain, data science, implementation, and regulatory requirements are required to develop ML solutions.^6^ Concerns about the robustness and generalizability of models applied to diverse populations, as well as the quality and accessibility of reporting ML methods and results, are growing as ML models in medicine are developed. The evaluations of bias, transparency, and reporting of ML research in a number of medical fields are unstandardized and amenable to improvement. Algorithmic bias (the representation of diversity in input data versus the target algorithm deployment population) is of particular concern for ML in medicine.^7^, ^8^ Previously, statistical clinical risk prediction models faced similar challenges, which were addressed by the creation of standardized analysis and reporting frameworks.^3^, ^9^, ^10^, ^11^ Similar frameworks are now being developed for clinical ML tools.^12^, ^13^ These novel frameworks must consider clinical utility and impact on both the patient and physician, as well as the rapidly evolving range of ML approaches and the data used to develop the models. Because of the variety of input data and the customizability of ML methods, disease‐ and domain‐specific recommendations are likely to be required for ML. While broad research and reporting guidelines are appropriate for more traditional prediction models, disease‐ and domain‐specific recommendations are likely to be necessary for ML.

In this study, we aimed to survey the spectrum of hypertension research employing ML, evaluate the quality of their reporting, and gain insight into the obstacles impeding the realization of ML's potential to transform hypertension care. Understanding where ML has been applied and its limitations will inform the design and reporting of future ML studies that can transform hypertension care.

## Methods

Our goal was to assess the topics covered in hypertension ML research and the current standard of communication of clinical ML research in hypertension using a custom survey developed by incorporating recommendations from existing checklists. The data that support the findings of this study are available from the corresponding author upon reasonable request. Institutional review board approval for this study was not required as this is a survey of published studies.

### Identification and Selection of Articles

A search was conducted across 3 widely used databases (Embase, PubMed, and Google Scholar) using 2 groups of medical subject headings search terms: those pertaining to hypertension (eg, “blood pressure,” “hypertension,” “ambulatory blood pressure monitoring”) and those pertaining to ML (eg, “machine learning,” “supervised machine learning,” “deep learning”). Non–medical subject headings search terms (eg, “random forest” and “Boltzmann machine”) were also included in the ML group. The inclusion criteria for search results were peer‐reviewed original research, publication date between January 2019 and September 2021, full text availability (either for free or via institutional access), and original English text. The articles were reviewed manually by separate teams at the Universities of Glasgow and Toledo. Selected articles were pooled, and those not meeting eligibility criteria were removed.

### Development of the Harmonious Understanding of Machine Learning Analytics Network Survey Questionnaire

A PubMed search identified ML reporting and evaluation frameworks published between January 2015 and February 2020. A group of ML specialists and hypertension researchers reviewed frameworks ranging from narrow domain‐specific to broader high‐level checklists.^3^, ^9^, ^12^, ^13^, ^14^ Based on this review, a list of survey items was generated and developed into the Harmonious Understanding of Machine Learning Analytics Network survey through an iterative Delphi process. The final survey contains 60 questions with binary, multiple choice, or free‐text responses (Table S1). Free‐text sections were included to provide additional comments or elaborate when responses like “Other” were selected in multiple‐choice questions.

### Survey Procedures

The Harmonious Understanding of Machine Learning Analytics Network survey was implemented in REDCap,^15^ which is a secure web application for building and managing online surveys. Two researchers (C.D.T. and T.Q.B.T.) read all the papers and completed the survey. In addition, 18 reviewers reflecting the typical readership of cardiovascular research journals also completed the survey. Reviewers were required to have experience with health care data but not with ML. Each article was reviewed by 2 randomly allocated reviewers who independently applied the Harmonious Understanding of Machine Learning Analytics Network survey to the article. Discordance was resolved with the opinion of a third reviewer with ML experience (C.D.T. or T.Q.B.T.). Responses were analyzed for each survey item. Adherence (ie, the proportion of articles that satisfied the questionnaire requirements) was calculated for each individual survey item. Qualitative results were grouped into 9 domains (clinical relevance; defining and addressing the knowledge gap [rationale]; prespecified study design; data suitability; ground truth [basis of supervised machine learning labeling]; performance metrics; replication and validation; ethical, legal, and social implications; and reporting quality). Data from REDCap were analyzed and visualized using the R programming language version 4.1.1 (R Foundation for Statistical Computing, Vienna, Austria).

## Results

**Figure 1:**
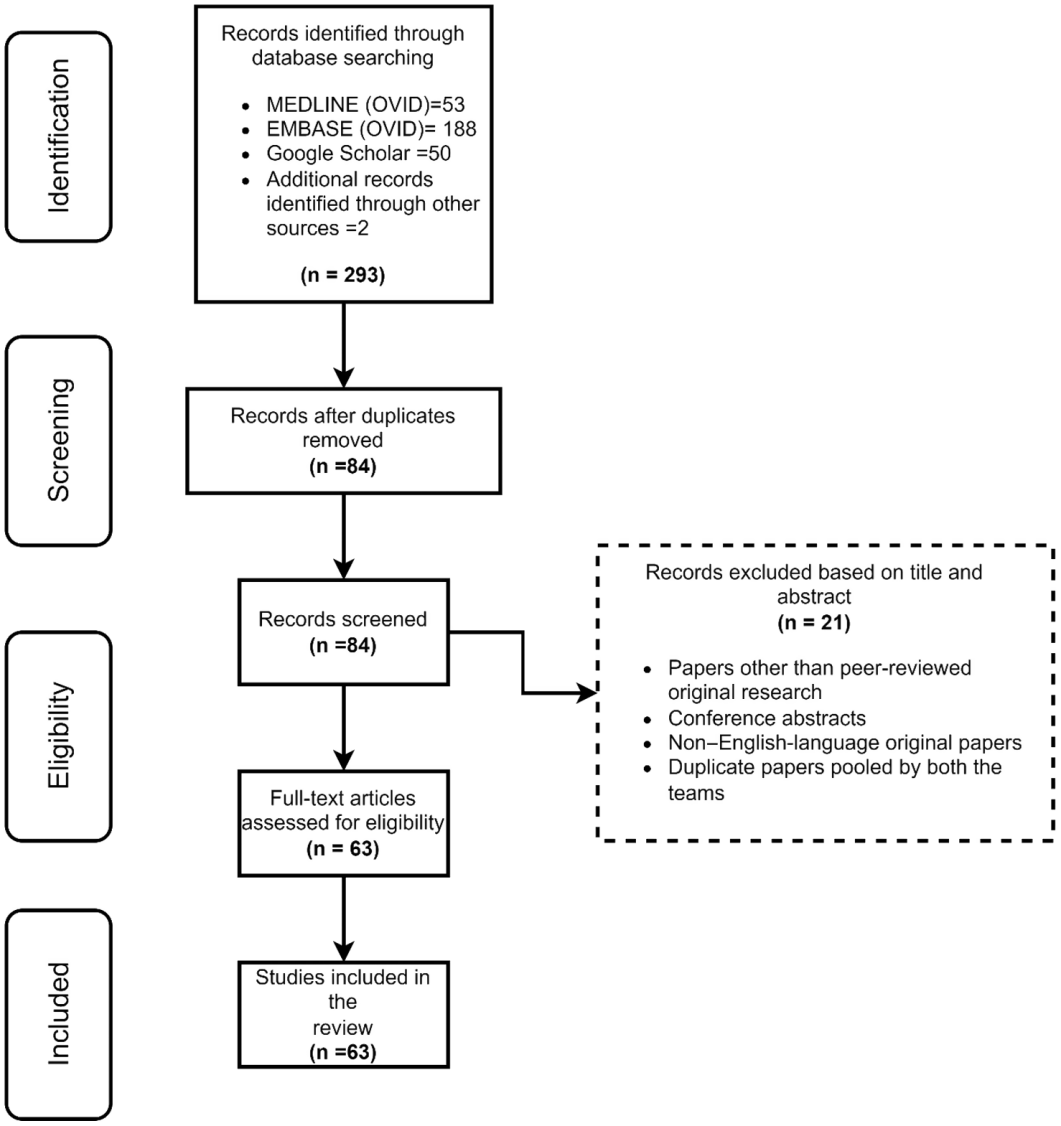
Preferred Reporting Items for Systematic Reviews and Meta‐Analyses flow diagram of the article screening and identification process.

**Figure 2:**
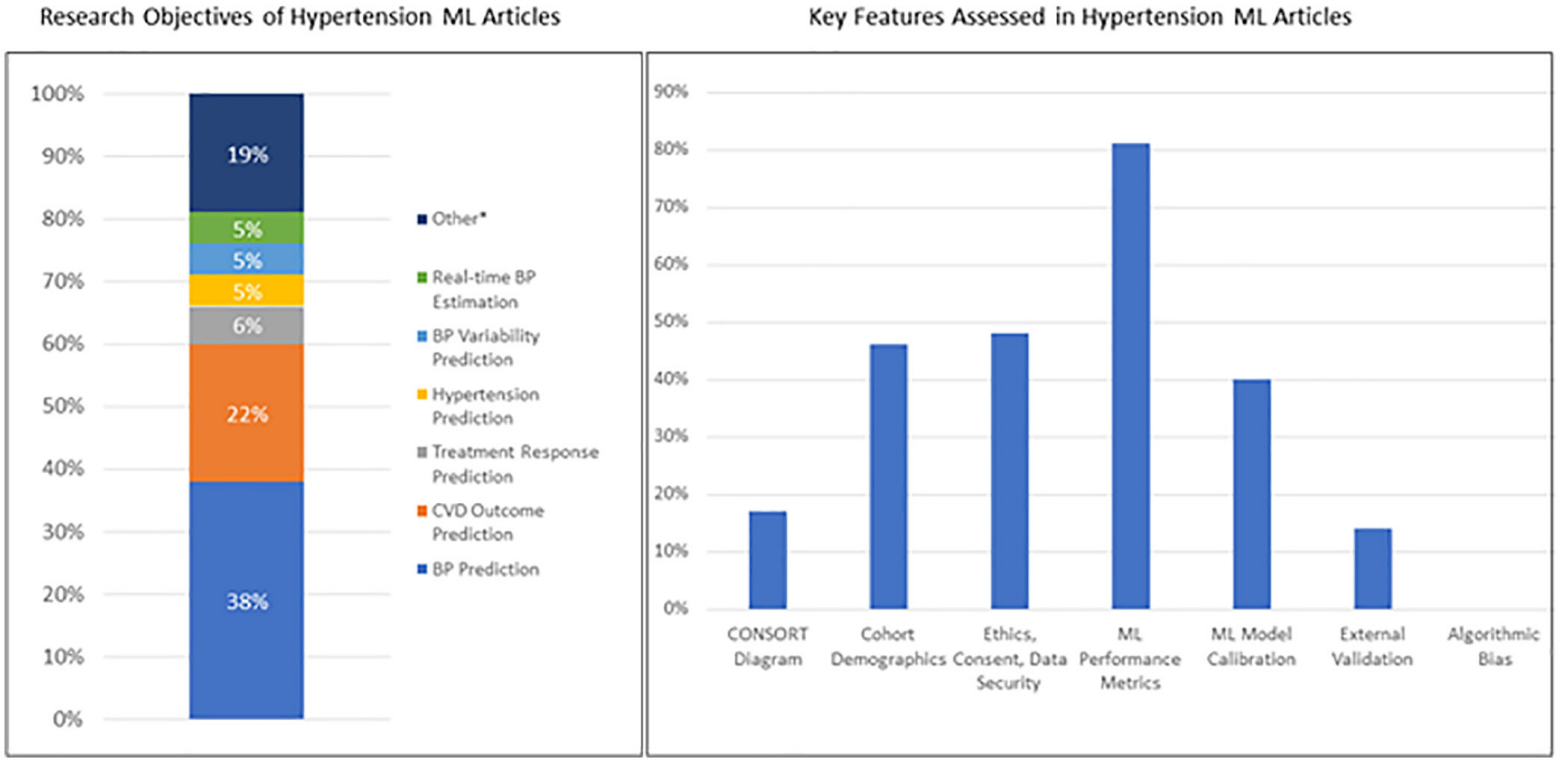
Research objectives of hypertension ML articles (left) and key findings of applying the Harmonious Understanding of Machine Learning Analytics Network survey to hypertension ML articles (right). *“Other” category includes objectives such as medication adherence, hypertension classification, risk stratification, and investigating the association of BP with the microbiome. BP indicates blood pressure; CONSORT, Consolidated Standards of Reporting Trials; CVD, cardiovascular disease; and ML, machine learning.

The search strategy identified 63 articles that applied ML in hypertension research. A Preferred Reporting Items for Systematic Reviews and Meta‐Analyses flow diagram outlining the selection process is presented in Figure 1. A list of the articles with main ML methods and objectives is presented in Table S2. The research objectives and data are summarized in Figure 2. The most frequent research aims were blood pressure (BP) prediction (38%), hypertension (22%), cardiovascular outcomes (6%), BP variability (5%), treatment response (5%), and real‐time BP estimation (5%).

The main data type used in each study was also identified. The most frequently used input data were routine clinical and demographic data retrieved from medical health records (44%). Thirty percent of studies chiefly used data from noninvasive methods, such as auscultatory or oscillometric BP measurements, photoplethysmography, or electrocardiography. Two of the 5 studies that reported using data from wearable devices did not specify how the measurements were made (eg, Apple watch uses photoplethysmography, but this was not specified).

### Survey Responses

All survey questions and responses are presented in Table S1.

### Traditional Components of Scientific Papers and Clinical Relevance

Fifty‐three of 63 articles (84%) described the relevance of their project in terms of clinical impact (potential savings in cost, lives, or time), and 89% described the rationale for the project and the knowledge gap being addressed. A notable exception to standard reporting requirements was the absence of a description of input data or cohort demographics in many articles (presented in 46% of articles).

### Prespecified Analysis Plan; Data; Validation; and Ethical, Legal, and Social Implications

In 59 of the 63 studies, the data sets used were deemed appropriate for the investigation, but in only 30 of the studies were the data obtained from the intended stage of the care pathway if the results were to be implemented. Most studies (44; 70%) also presented a prespecified statistical analysis plan, and 63 studies explained data preprocessing and curation steps.

Internal validation methods, such as cross‐fold validation or use of independent training and testing data sets, were described in 73% of studies. External validation with geographically or temporally distinct data sets was carried out in 9 (14%) studies.

Compliance with ethical, patient privacy, and data security regulations were mentioned in 30 (48%)  articles. Of the 39 prospective or interventional trials that were deemed by reviewers to require informed consent, acquiring patient consent was mentioned in 17 (44%) studies. Algorithmic bias was not rigorously addressed in any of the reviewed studies, with only 6 articles acknowledging a risk of bias.

### Ground Truth and Performance Metrics

Almost all of the studies (58; 92%) applied supervised learning techniques requiring the establishment of ground truth for analysis. Ground truth labels in 47 of the 58 studies (81%) were sufficiently explained and backed by guidelines or references.

Most articles (51; 81%) reported at least 1 model performance measure (eg, accuracy, sensitivity, or area under the receiver operating characteristic curve). In contrast, a minority (40%) presented any calibration measures (eg, calibration plot, Hosmer–Lemeshow test, or Brier scores), and 37 of 63 studies described measures to address overfitting.

### General Readership Survey

**Figure 3:**
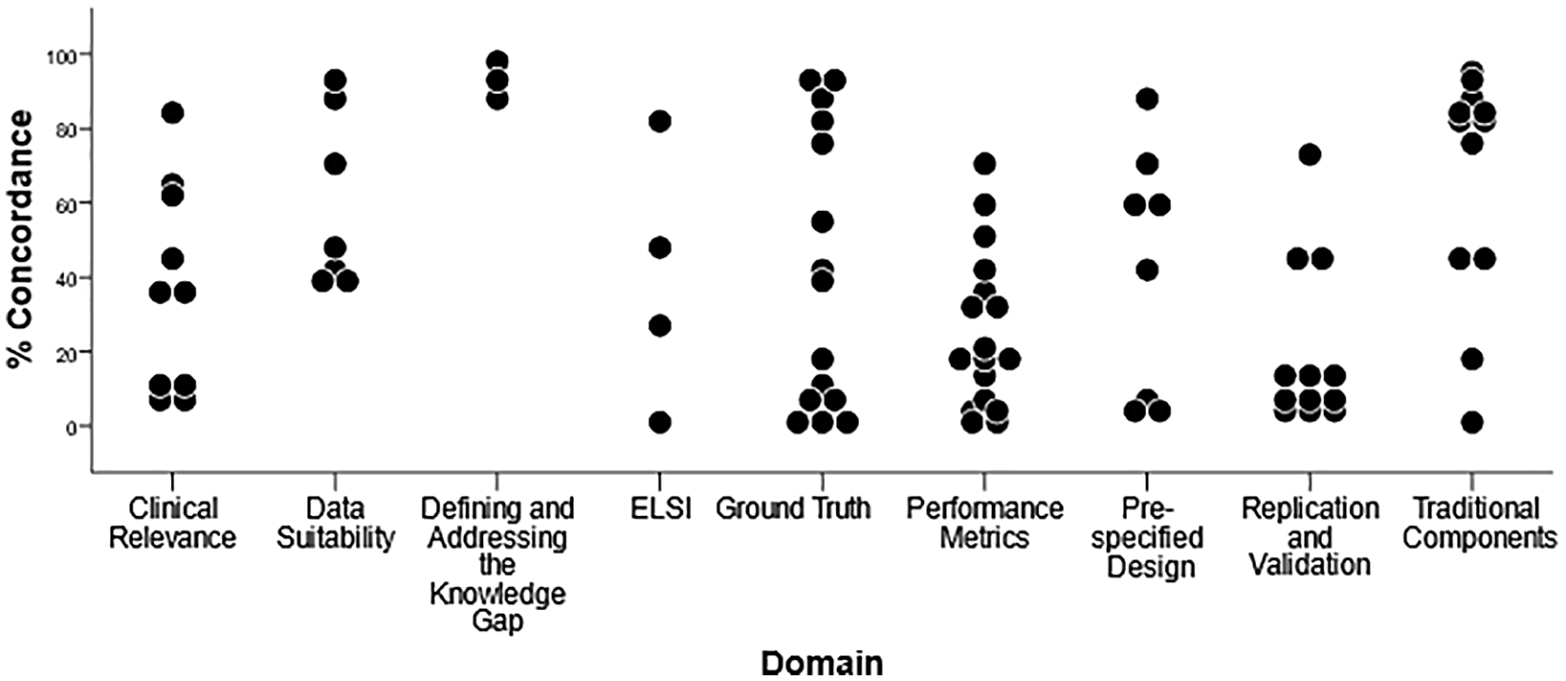
Dot plot showing percentage concordance between non–machine learning expert reviewers, representing real‐world readership of the research articles. Each dot represents 1 question, and its position on the *y* axis represents the concordance between pairs of reviewers. ELSI indicates ethical, legal, and social implications.

Figure 3 shows the percentage of concordance between non‐ML expert reviewers representing real‐world readership of the research articles. The highest concordance was seen for items with which the readership is expected to be familiar (namely, general publication quality questions). Lower concordance was observed for questions that covered technical clinical or ML aspects; for example, only 50% of reviewers agreed with their counterpart when assessing items related to overfitting.

## Discussion

Our survey of hypertension‐related publications over a 33‐month period showed that ML use is limited to exploratory research and has significant shortcomings in reporting quality, model validation, and algorithmic bias. Our analysis identifies areas for improvement that will facilitate the full realization of the potential of ML in hypertension and facilitate its adoption.

The most common research topics were BP prediction, hypertension, and cardiovascular risk, all of which are unquestionably important; however, most of the studies were exploratory and have low translational potential due to the need for multiple validations in independent data sets and long follow‐up for definitive outcomes. Successful applications of ML include the automation of tasks and the management of chronic diseases such as hypertension. These may be the “low‐hanging fruit” of implementable ML for the clinical management of hypertension, and studies examining adherence, managing follow‐up, monitoring home BP, risk factor management, treatment titration, and education may yield simple solutions that could revolutionize hypertension care.

ML research imposes additional requirements on its design, execution, and reporting that are essential for establishing confidence in novel applications and accelerating their clinical implementation for the benefit of patients. The reporting must be of high quality to demonstrate scientific rigor and should be understandable to a reader who may not be an expert in ML. The engagement of domain experts is crucial, as they are the source of clinical challenges that ML specialists must address.

Using the most suitable data for the research question is crucial to algorithm development. In both prospective and retrospective medical research, best practices, epidemiological research, and other earlier works typically guide the selection of study population and outcome. Frameworks such as Population, Intervention, Comparator, and Outcomes provide guidance on formulating the research question and implementing best practices in clinical research.^10^

Most reviewed studies (89%) employed data sets deemed suitable for the clinical question being investigated. In nearly half of the articles, data selection criteria and study populations were not described. Likewise, 44% of studies lacked adherence to transparency and ethics. It is possible that studies followed regulations but did not explicitly document it.

Presenting data appropriately is essential to convince readers that all efforts were taken to minimize bias.^11^ It clarifies the populations to which the study's findings are applicable, which could aid in the future implementation of new interventions or algorithms. Algorithmic bias was the survey item that appeared the least frequently in the articles. Algorithmic bias refers to the extent to which diversity (eg, racial, socioeconomic, sex, and age) is present in the data set used for model development versus the deployment population.^8^ Biases in the model's training data may be propagated through its development and eventual deployment, thereby fostering greater inequality. Systematic bias and fairness testing is the first step in informed model selection, which reduces ML‐caused inequities.

The most common ML technique in the articles reviewed was supervised learning. As supervised learning depends on models learning from labeled examples, the quality of the ground truth (on which the labels are based) is crucial. Without meticulously selected and labeled data, models cannot be effectively constructed or evaluated. Existing guidelines supported the majority of studies' ground truth labeling, lending credibility to the performance of the resulting models. Studies reported a variety of model performance metrics, but the selection of metrics should be appropriate for the model and the clinical setting in which it will be used.

For prediction models, calibration and discrimination are the minimum requirements for reporting,^2^ and only a minority of articles reported calibration. The area under the precision‐recall curve should be reported alongside area under the receiver operating characteristic curve metrics for imbalanced data, for which area under the receiver operating characteristic curve metrics were typically reported. Additionally, accuracy and harmonic mean of precision and recall score should be reported, the latter especially when the data set is unbalanced.^2^, ^14^

Most articles viewed overfitting as a threat to the validity of their models. Studies must consider the risk of overfitting as well as countermeasures (eg, oversampling or undersampling). Downsampling is inefficient because reducing the sample size may increase the likelihood of overfitting.^14^ Root mean squared error or mean absolute error is recommended for continuous variables. In addition to sample size, number of predictors, and hyperparameter tuning, other factors that influence differences in performance and must therefore be described in detail are sample size, number of predictors, and variance in performance. In varying degrees, these requirements were met in the studies surveyed.

External validation (in geographically or temporally distinct training and validation data sets) is essential before clinical implementation to demonstrate accuracy and generalizability in settings and populations beyond the original derivation population. Typically, external validation studies are anticipated to diminish the predictive accuracy of models. Only 5 studies reported validating the ML model against an external data set in our review. This may be due to a lack of appropriate external data sets or lack of awareness of the importance of external validation. Another explanation may be the belief that splitting the data set into training and testing sets satisfies the need for validation. Here, we stress the importance of having a totally separate test data set or sometimes several separate test sets, with hyperparameter fine‐tuning carried out using a validation data set. One needs to be careful with hyperparameter optimization because changing hyperparameters changes the performance of the whole model and may overfit to the peculiarities of the validation set; cross validation may help to some extent, but an independent test set is the ideal solution.

The clinical usefulness, trustworthiness (to both patients and physicians), and explainability of an algorithm all contribute to its clinical adoption. As a result, providing a detailed description of how the proposed ML model aligns on these dimensions would be beneficial for eventual implementation. If applicable to the stage of the study, plans for deployment and commercialization, including regulatory requirements, may need to be considered. Patients and the general public should be involved in research, and there should be a clear strategy in place to evaluate the acceptability of the proposed model and outcomes to the patients providing the data, the clinicians applying the models, and the patients to whom the model will be applied.

The current study has some limitations. First, it is a scoping review, and while every effort was made to capture the full spectrum of publications in the cross section of ML and hypertension research, individual articles may have been overlooked. Second, the Harmonious Understanding of Machine Learning Analytics Network survey omitted some critical ML‐related questions, such as data availability, code sharing, transparency, explainability, and interpretability of ML models.

Finally, with the increasing use of ML methods in hypertension research, our analysis of recent hypertension ML publications identifies areas for improvement in reporting, which should inform and support hypertension researchers who are using or planning to use ML. This will ensure that ML research in hypertension satisfies the global consensus that ML solutions must be fair and nondiscriminatory, while also having a positive impact in all areas of social and economic life.

## Sources of Funding

Dr Padmanabhan is supported by the British Heart Foundation Centre of Excellence Award (RE/18/6/34217) and the United Kingdom Research and Innovation Strength in Places Fund (SIPF00007/1). T.Q.B. Tran is supported by a British Heart Foundation MBPhD Studentship (FS/MBPhD/22/28005). R01HL143082 funding from the National Heart, Lung, and Blood Institute of the National Institutes of Health to B. Joe is gratefully acknowledged.

## Disclosures

None.

## Supporting information

Tables S1–S2
References 17–78
