# Supplementary material for: Survey and Evaluation of Hypertension Machine Learning Research
Source: J Am Heart Assoc. 2023 Apr 29;12(9):e027896. doi: 10.1161/JAHA.122.027896 (PMC10227215; doi:10.1161/JAHA.122.027896)
Supplement: Supplementary file 1 — Tables S1–S2 References 17–78 [file JAH3-12-e027896-s001.pdf]

Supplemental Material

Table S1: Results from the application of the HUMANE checklist to articles included in analysis. Each question shows the total number of papers that were scored for each choice. The responses are adjudicated responses, where the two main authors CDT and TQBT resolved any discordant responses to a single response.

Question		Options		Responses	Response (%)
**Clinical Relevance**					
**Q1**	**Is the importance of research (e.g., cost/life/time/process savings) explained?**	Yes		53	84%
		No		10	16%
**Q2**	**Which of the following domain(s) did the article explore for potential impact of the model? (check all that apply)**	Triage	Checked	4	6%
			Unchecked	59	94%
		Early Diagnosis	Checked	23	37%
			Unchecked	40	63%
		Improved Diagnosis	Checked	29	46%
			Unchecked	34	54%
		Allowed personalized/targeted treatment	Checked	6	10%
			Unchecked	57	90%
		Prevent/reduce hospital admissions	Checked	5	8%
			Unchecked	58	92%
		Improve survival	Checked	6	10%
			Unchecked	57	90%
		Other	Checked	22	35%
			Unchecked	41	65%
**Q3**	**Is the intended role of the model (e.g., triage or diagnosis) clear?**	Yes		41	65%
		No		6	10%
		NA		16	25%
**Q4**	**Is it clear whether the model be used as an isolated test or in combination with other diagnostic elements?**	Yes		39	62%
		No		11	17%
		NA		13	21%
**Defining and Addressing the Knowledge Gap**					
**Q1**	**Have the authors detailed what is already known in the field?**	Yes		62	98%
		No		1	2%
**Q2**	**Is the knowledge gap defined?**	Yes		56	89%
		No		7	11%
**Q3**	**Have the authors explained how they aim to address the knowledge gap?**	Yes		59	94%
		No		4	6%
**Pre-specified Study Design**					
**Q1**	**Is the experimental protocol designed to prevent overfitting?**	Yes		37	59%
		No		22	35%
		NA		4	6%
**Q2**		Yes		27	43%

	**Are there pre-defined inclusion and exclusion criteria for different model/study datasets?**	No		27	43%
		NA		9	14%
**Q3**	**Does the outcome tested by the ML model align with written methods?**	Yes		56	89%
		No		6	10%
		NA		1	2%
**Q4**	**Has the study described any other multivariable prediction models?**	Yes		38	60%
		No		20	32%
		NA		5	8%
**Q5**	**Has the study pre-specified a statistical analysis plan?**	Yes		44	70%
		No		19	30%
**Q6**	**Has the study applied any of the following methods to address class imbalance?**	Oversampling - adding copies of underrepresented class		4	6%
		Undersampling - removing copies of overrepresented class		3	5%
		Replicate the class distribution in the validation test set		3	5%
		Other			
		None Reported		53	84%
**Data Suitability**					
**Q1**	**Is the study methodology and study pre-specified in terms of the study design (e.g., retrospective/prospective, derivation/validation, supervised/unsupervised/deep learning), including characteristics of the data type collected?**	Yes		55	87%
		No		8	13%
**Q2**	**Is the study timeline specified in terms of initiation of data collection/model development and the end date of the completed (or ongoing) data collection/model validation?**	Yes		26	41%
		No		37	59%
**Q3**	**Is the dataset obtained from within the intended stage in the care pathway?**	Yes		30	48%
		No		5	8%
		Unclear		28	44%
**Q4**	**Are the key data pre-processing/pre-curation steps described?**	Yes		45	71%
		No		18	29%
**Q5**	**Is the dataset appropriate for the healthcare conditions studied?**	Yes		59	94%
		No		4	6%
**Q6**	**Is there sufficient clarity on dataset for model development (training/test/validation)?**	Clear		24	38%
		Partially Clear		25	40%
		Unclear		14	22%
**ELSI**					
**Q1**	**Is it explicitly mentioned that study is compliant with local ethical**	Yes		30	48%
		No		27	43%

	**committee/IRB/patient privacy/data security regulations?**	NA		6	10%
**Q2**	**Has documented consent been obtained from the participants involved in the prospective/intervention study?**	Yes		17	27%
		No		22	35%
		NA		24	38%
**Q3**	**Has the article evaluated algorithmic bias? (e.g., gender, race, ethnicity, socioeconomic status etc.)**	Yes		1	2%
		No		57	90%
		Partial		5	8%
**Q4**	**Have the authors listed their conflict of interest(s)?**	Yes		51	81%
		No		12	19%
**Ground Truth**					
**Q1**	**Is ground truth applicable for supervised learning method in this article?**	Yes		58	92%
		No		5	8%
**Q2**	**How much do you agree with the accuracy of the ground truth labels (is labelling backed by clinical guidelines or references; are sufficient details provided on the ground truth labelling process)?**	Strongly Agree		22	38%[a]
		Agree		25	43%[a]
		Neutral		10	17%[a]
		Disagree		0	0%[a]
		Strongly Disagree		1	2%[a]
**Q3**	**Were ground truth labels manually determined by experts?**	Yes		32	55%[a]
		No		26	45%[a]
**Q4**	**Were ground truth labels automatically generated?**	Yes		7	12%[a]
		No		51	88%[a]
**Q5**	**Were any ground truth labels missing?**	Yes		0	0%[a]
		No		58	100%[a]
**Q6**	**How were the ground truth labels added?**	Prospectively		47	81%[a]
		Retrospectively		11	19%[a]
**Q7**	**Which of the following is applicable for the number of experts involved in the review?**	Single		54	93%[a]
		Multiple Independent		4	7%[a]
		Use of Adjudicator(s)		0	0%[a]
**Q8**	**Which of the following is applicable regarding the qualification of the expert(s) in the review?**	Sub-specialist with experience		4	7%[a]
		Board-certified specialist		1	2%[a]
		Specialist in the domain without sub-specialty accreditation		0	0%[a]
		Others		53	91%[a]
**Q9**	**Was there sufficient availability of clinical information to the expert to make the diagnosis?**	Yes		48	83%[a]
		No		0	0%[a]
		Unclear		10	17%[a]
**Q10**	**Is an inter-observer agreement presented?**	Yes		0	0%[a]
		No		4	7%[a]
		NA		54	93%[a]
**Performance Metrics**					
**Q1**		Yes		22	35%

	**Was the distribution of outcomes similar in all training, test and validation datasets?**	No		6	10%
		NA		35	56%
**Q2**	**Has the study specified a range of statistical measures used to compare the accuracy/precision/sensitivity/specificity of the proposed model?**	Yes		44	70%
		No		19	30%
**Q3**	**Has the article presented any difference between the training, testing, and validation data sets in inclusion criteria, model outcome, and predictors?**	Yes		8	13%
		No		39	62%
		NA		16	25%
**Q4**	**Has the study reported any discrimination measures of performance? (Check all that apply)**	Accuracy	Checked	32	51%
			Unchecked	31	49%
		Sensitivity/Recall	Checked	20	32%
			Unchecked	43	68%
		Specificity	Checked	12	19%
			Unchecked	51	81%
		Precision	Checked	13	21%
			Unchecked	50	79%
		ROC curve	Checked	20	32%
			Unchecked	43	68%
		Precision recall (PR) curve	Checked	3	5%
			Unchecked	60	95%
		Other	Checked	26	41%
			Unchecked	37	59%
		None reported	Checked	12	19%
			Unchecked	51	81%
**Q5**	**Has the article reported any calibration measures of performance? (Check all that apply)**	Calibration plot	Checked	4	6%
			Unchecked	59	94%
		Hosmer-Lemeshaw test	Checked	1	2%
			Unchecked	62	98%
		Excepted calibration error	Checked	0	0%
			Unchecked	63	100%
		Brier score	Checked	2	3%
			Unchecked	61	97%
		Mean square error (MSE)	Checked	11	17%
			Unchecked	52	83%
		Other	Checked	12	19%
			Unchecked	51	81%
		None reported	Checked	38	60%
			Unchecked	25	40%
**Replication and Validation**					
**Q1**	**Is the validation dataset distinct from training and test datasets?**	Temporally		3	5%
		Geographically		2	3%
		Both		5	8%
		None		53	84%

**Q2**	**Has the study described the predictor model using an internal validation technique?**	Yes		46	73%
		No		11	17%
		NA		6	10%
**Q3**	**How was the experimental protocol developed to prevent overfitting?**	Independent train and test dataset validation		5	8%
		Crossfold validation		29	46%
		Leave one out validation		3	5%
		Other		0	0%
		Not Applicable (NA)		26	41%
**Q4**	**Was model validation performed using an out-of-sample external validation dataset?**	Yes		9	14%
		No		54	86%
**Q5**	**What other steps are reported to support external validity?**	Disease prevalence in the internal validation test dataset representative of the target population in the real world		9	14%
		Presence of subgroups within the training dataset		5	8%
		Authors have not applied any inclusion or exclusion criteria which create a selection bias		28	44%
		Authors have applied a sampling method (i.e. random sampling) to reduce the risk of spectrum bias?		8	13%
		Other		13	21%
**Traditional components of scientific papers**					
**Q1**	**Is the title relevant to research in the field of AI/ML in medicine?**	Yes		52	83%
		No		11	17%
**Q2**	**Does the title align with any of the following terms or related terms: AI, ML, or deep learning?**	Yes		55	87%
		No		8	13%
**Q3**	**Does the abstract provide a summary of the following: objectives, study design, setting, target population, statistical analysis, results, and conclusion pertinent to ML in healthcare?**	Agree		29	46%
		Partially Agree		26	41%
		Disagree		8	13%
**Q4**	**Has the article defined the objectives including validation or development of ML?**	Yes		53	84%
		No		10	16%
**Q5**	**Is there a pre-specified threshold for inclusion of cases where there is non-consensus?**	Yes		1	2%
		No		20	32%
		NA		42	66%

**Q6**	**Has the study described key demographics/characteristics of the cohorts? (Table 1- age, gender, chronic co-morbidities, patient type etc.)**	Yes		29	46%
		No		34	54%
**Q7**	**Has the study described either in text or by a flow diagram the impact of applying stated inclusion/exclusion criteria on the final sample size?**	Yes		11	17%
		No		52	83%
**Q8**	**Has the study provided a succinct summary of their primary result findings?**	Yes		60	95%
		No		3	5%
**Q9**	**Has the study compared their results with existing literature, by supporting or challenging their findings?**	Yes		52	83%
		No		11	17%
**Q10**	**Has the article mentioned strengths of their research?**	Yes		53	84%
		No		10	16%
**Q11**	**Has the article mentioned weaknesses of their research?**	Yes		48	76%
		No		15	24%
**Q12**	**Have the authors provided a justifiable conclusion based on the results presented with a take-home message and implications of the results?**	Yes		59	94%
		No		4	6%

[a] These percentages are out of 58, the number of 'Yes' responses to Ground Truth Q1.

Table S2: Articles included in analysis. 4D MRI: 4-dimensional magnetic resonance imaging; ANN: Artificial Neural Network; BiLSTM: Bidirectional LSTM; BP: blood pressure; CART: Classification And Regression Trees; CNN: Convolutional Neural Network; DANN: Domain-Adversarial Training of Neural Networks; DBN: Deep Belief Network; DNN: Deep Neural Network; ECG: electrocardiogram; GNN: Graph Neural Network GPR: Gaussian process regression; HTN: hypertension; KNN: k-Nearest Neighbors; LASSO: Least Absolute Shrinkage and Selection Operator; LDA: Linear Discriminant Analysis; LightGBM: Light Gradient Boosting Machine; LSTM: Long Short-Term Memory networks; LSVM: Lagrangian Support Vector Machine; ML: machine learning; MLP: Multilayer perceptron; MNN: Modular Neural Network; NBC: Naive Bayes Classifier; PPG: photoplethysmography; RCT: Randomised Controlled Trial; RF: Random Forest; RFE: Recursive Feature Elimination; RL: Reinforcement Learning; RNN: Recurrent Neural Network; SOM: Self-Organizing Map; SVM: Support Vector Machines; SVR: Support Vector Regression.

**Publication**	**Data source**	**ML task**	**ML methods and study objectives**	**Ref.**
Aziz et al. 2020	Adherence questionnaire, demographics, medical records	Drug adherence	Use ML (RF ANN, SVR, SOM) to find determinants of antihypertensive medication adherence & predict precise adherence scores.	16
Argha et al. 2019	Auscultatory waveforms	Predict BP	Use DL (LSTM-RNN) to estimate SBP & DBP from auscultatory waveforms.	17
Argha et al. 2021	Auscultatory waveforms	Predict BP	Use DL (BiLSTM-RNN) to estimate SBP & DBP from auscultatory waveforms.	18
Pan et al. 2019	Auscultatory waveforms	Predict BP	Use ML (CNN) to determine BP from Korotkoff sound recordings.	19
Pan et al. 2019	Auscultatory waveforms	Predict BP	Use ML (CNN) to determine impact of movement disturbance on BP measurement.	20
Persell et al. 2020	Medical records (clinical trial)	HTN management	AI based coaching app for HTN management.	21
Miao et al. 2020	ECG	Predict BP	Use ML (CNN with LSTM) to estimate BP from ECG data.	22
Soh et al. 2020	ECG	Predict BP	Use ML (k-NN, decision tree, LDA) to identify masked HTN from ECG data without ABPM.	23
Li et al. 2020	ECG & PPG	Predict BP	Use ML (LSTM) to estimate BP from PPG &E CG signals in real time.	24
Yan et al. 2019	ECG & PPG	Predict BP	Use ML (CNN) to estimate BP from PPG & ECG signals in real time.	25

Zhang et al. 2019	ECG & PPG	Predict BP	Use ML (SVR) to estimate BP PPG & ECG signals & other physiological measurements.	26
Sannino et al. 2020	ECG & PPG	Predict HTN	Comparison of discriminative performance of several ML models (in classifying HTN from PPG & ECG data).	27
Li et al. 2019	Genetic data	Predict HTN	Use ML (SVM) to predict HTN from genetic & environmental risk factors.	28
Widen et al. 2021	Genetic data & medical data	Predict BP	Use ML (LASSO) to predict quantitative traits from genomic data	29
Kissas et al. 2020	Imaging, computational fluid dynamics, 4D MRI	Predict BP	Use physics informed neural networks to predict BP from 4D flow MRI	30
Lacson et al. 2019	Medical records	BP variability	Use ML (random forest) to identify features affecting SBP variability.	31
Barbieri et al. 2019	Medical records	BP, fluid management and dialysis	Use ML (ANN) to guide BP, fluid volume & dialysis dose in ESKD	32
Cho et al. 2020	Medical records	CVD/ outcomes	Use DL (RNN-LSTM) & Cox regression to predict CVD.	33
Du et al. 2020	Medical records	CVD/ outcomes	Use ML (XGBoost, kNN, SVM, decision tree, random forest) & logistic regression to predict CHD risk factors.	34
Wu et al. 2019	Medical records	CVD/ outcomes	Use ML (ANN) to predict NSTEMI.	35
Wu et al. 2020	Medical records	CVD/ outcomes	Use ML (XGBoost) to predict outcomes of young patients with HTN.	36
Bertsimas et al. 2021	Medical records	Personalised treatment	Use ML (ensemble of multiple methods) to personalise ACEI/ARB treatment for hypertensive COVID-19 patients.	37
Zheng et al. 2021	Medical records	Predict BP	Use ML (SVM, decision tree, GPR, ANN, logistic regression) to predict SBP from clinical features.	38
AlKaabi et al. 2020	Medical records	Predict HTN	Use supervised ML models (decision tree, random forest, logistic regression) to predict hypertension from 987 biobank records.	39
Chang et al. 2019	Medical records	Predict HTN	Use ML (SVM, decision tree, random forest, XGBoost) to predict HTN from clinical data.	40
Elshawi et al. 2019	Medical records	Predict HTN	Use ML (random forest) to predict hypertension risk from fitness data & evaluate interpretability.	41

Fang et al. 2021	Medical records	Predict HTN	Use ML (k-NN, LightGBM, SVM, random forest) to predict 5-year HTN risk from medical records.	42
Islam et al. 2021	Medical records	Predict HTN	Use ML (ANN, decision tree, random forest, gradient boosting) to characterise HTN risks (features identified with LASSO & SVM RFE).	43
Kanegae et al. 2020	Medical records	Predict HTN	Use ML (XGBoost & ensemble model) for hypertension risk prediction.	44
López-Martínez et al. 2020	Medical records	Predict HTN	Use ML (ANN) to predict HTN from demographic & clinical features.	45
Marin et al. 2019	Medical records	Predict HTN	Use ML (random forest, SVM, Gaussian Naïve Bayes, logistic regression) to classify hypertension from medical data.	46
Nour et al. 2020	Medical records	Predict HTN	Use ML (random forest, decision tree, LDA, LSVM) to classify hypertension from medical data.	47
Xu et al. 2019	Medical records	Predict HTN	Use ML (ANN, NBC, CART) to predict HTN risk (development & validation of population-specific HTN risk prediction model).	48
Diao et al. 2021	Medical records	Predict secondary HTN	Use ML (XGBoost) to predict aetiology of secondary HTN.	49
Boutilier et al. 2021	Medical records	Risk stratification	Use ML (decision tree, random forest, RL, k-NN, AdaBoost) for risk stratification of HTN & diabetes in resource-limited LMICs.	50
Chunyu et al. 2020	Medical records	Treatment effects	Use ML (LASSO, mean decrease impurity, recursive feature elimination, ensemble models) to find features contributing to treatment response to 5 commonly prescribed anti-HTN drugs.	51
Angelaki et al. 2021	Medical records & ECG	Predict LVH	Use supervised ML (random forest) to detect abnormal LVG before onset of LVH from ECG & basic clinical parameters from 528 normotensive & hypertensive patients.	52
Gupta et al. 2021	Medical records & imaging	Predict HTN in pregnancy	Use ML (CNN) to predict HTN from placental ultrasound images in pregnancy.	53
Koshimizu et al. 2020	Medical records (clinical trial)	BP variability	Use ML (DNN) to predict BP variability from PREDICT trial data.	54
Esmaelpoor et al. 2020	Medical records, PPG	Predict BP	Use DL (DNN) to estimate BP from PPG.	55
Liu et al. 2020	Nutritional data	Predict HTN	Use ML (SVM, decision tree, random forest, MLP, XGBoost) to predict HTN from nutritional intake.	56

Verhaar et al. 2020	Nutritional, microbiome data	Predict BP	Use ML (XGBoost) to investigate association of microbiome & BP.	57
Alghamdi et al. 2020	Oscillometric waveforms	Predict BP	Use supervised ML models (kNN, WkNN, bagged trees) to predict SBP & DBP from oscillometric waveforms from 350 patients.	58
Argha et al. 2020	Oscillometric waveforms	Predict BP	Use DL (LSTM-RNN) to estimate SBP & DBP from oscillometric waveforms.	59
Argha et al. 2019	Oscillometric waveforms	Predict BP	Use DL (DBN-DNN) to estimate SBP & DBP from oscillometric waveforms.	60
Celler et al. 2020	Oscillometric waveforms	Predict BP	Use ML (GMM-HMM) to estimate SBP & DBP from oscillometric waveforms.	61
Magbool et al. 2021	Other (simulated data)	Aortic BP	Use ML (decision tree, random forest, MLR, neural networks) to estimate aortic BP from simulated pulse wave dataset.	62
Singh et al. 2021	Other (unclear)	HTN, ABPM	Use ML (random forest) to predict HTN from clinical features	63
Pulido et al. 2019	Other (unclear)	Predict HTN	Use ML (MNN) to classify HTN from BP data.	64
Chowdhury et al. 2020	PPG	Predict BP	Use ML (SVR, GPR, regression trees, ensemble trees) & linear regression to determine BP from PPG.	65
Fujita et al. 2019	PPG	Predict BP	Use partial least-squares regression to estimate BP from PPG.	66
Maher et al. 2021	PPG	Predict BP	Use ML (SVM, ANN) to estimate BP from PPG.	67
Mejía-Mejía et al. 2021	PPG	Predict BP	Use ML (k-NN, SVM, ANN) to classify HTN and predict BP from PPG.	68
Chen et al. 2019	Pulse transit time	Realtime BP	Use ML (SVR) to continuously monitor BP from pulse transit time measurements.	69
Huttunen et al. 2019	Pulse transit time, simulated data	BP, aortic BP	Train ML model (Gaussian process regression) on simulated patient data for BP prediction from PTT.	70
Duan et al. 2019	Medical records (clinical trial)	Treatment effects	Use ML (X-learner) & logistic regression to predict treatment effect size of intensive & standard anti-HTN therapy.	71
Tsoi et al. 2020	Medical records (clinical trial)	BP variability	Use ML (K-means clustering, Partitioning Around Medoids, spectral clustering, Ward's method, Expectation Maximization) to cluster BP variability into groups.	72

Ankışhan et al. 2020	Speech recordings	Predict BP	Use ML (CNN, SVM/SVR, MLR) to predict BP from speech recordings from 86 subjects.	73
Chiang et al. 2019	Wearable technology	Personalised treatment	Use ML (random forest) to predict BP from wearable tech data & historical BP readings.	74
El Attaoui et al. 2021	Wearable technology	Realtime BP	Present a wireless medical sensor network with wireless BP sensing and ML (decision tree, kNN, NBC) to monitor BP in real time (for both patients & physicians).	75
Huang et al. 2019	Wearable technology	Realtime BP	ML (random forest, gradient boosting, adaptive boosting regression models) with wearable pulse wave sensor	76
Guthrie et al. 2019	Wearable technology	Treatment effects	Use ML (random forest) to develop digital biomarkers for digital therapeutic treatment response.	77
Zhang et al. 2020	Wearable technology, bioimpedance	Predict BP	Use ML (DANN) to estimate beat-to-beat BP from 5mins of bioimpedance data.	78
